# Insertion sequence elements-mediated structural variations in bacterial genomes

**DOI:** 10.1186/s13100-018-0134-3

**Published:** 2018-08-29

**Authors:** Etienne Nzabarushimana, Haixu Tang

**Affiliations:** School of Informatics, Computing and Engineering, Indiana University, Bloomington, IN USA

**Keywords:** Insertion sequence elements, Structural variations, Mutation accumulation, Bacterial genomes

## Abstract

Mobile genetic elements (MGEs) impact the evolution and stability of their host genomes. Insertion sequence (IS) elements are the most common MGEs in bacterial genomes and play a crucial role in mediating large-scale variations in bacterial genomes. It is understood that IS elements and MGEs in general coexist in a dynamical equilibrium with their respective hosts. Current studies indicate that the spontaneous movement of IS elements does not follow a constant rate in different bacterial genomes. However, due to the paucity and sparsity of the data, these observations are yet to be conclusive. In this paper, we conducted a comparative analysis of the IS-mediated genome structural variations in ten mutation accumulation (MA) experiments across eight strains of five bacterial species containing IS elements, including four strains of the E. coli. We used GRASPER algorithm, a *denovo* structural variation (SV) identification algorithm designed to detect SVs involving repetitive sequences in the genome. We observed highly diverse rates of IS insertions and IS-mediated recombinations across different bacterial species as well as across different strains of the same bacterial species. We also observed different rates of the elements from the same IS family in different bacterial genomes, suggesting that the distinction in rates might not be due to the different composition of IS elements across bacterial genomes.

**Electronic supplementary material:**

The online version of this article (10.1186/s13100-018-0134-3) contains supplementary material, which is available to authorized users.

## Main text

The intricacies of mobile genetic elements (MGEs) in their host genomes have challenged if not inspired scientists to understand the evolution and stability of genomes. MGEs can replicate from one location to another within a genome or between genomes (transposition) [1, 2], which is a major cause of large-scale genome reorganization in both eukaryotes and prokaryotes [1]. MGEs and their hosts typically have opposing interests (i.e., selection on MGEs favors elements with greater proliferative ability, whereas selection on the host favors less transposition meaning host selection acts on maintaining a coherent and functional genome and transposition would affect this), which define the co-evolution between both players and ultimately shape the architecture of host genomes [3]. Though generally deleterious, MGEs can ultimately contribute to the innovation of biological functions in the host genomes [4–10]. While the impact of MGEs on higher eukaryotic genomes is being increasingly recognized (e.g., the connection between MGEs and human diseases [11]), the studies of MGEs in bacteria and other lower organisms are relatively limited [2, 12, 13].

Insertion sequence (IS) elements are the smallest but a common class of MGEs in bacterial genomes. IS elements play a crucial role in mediating large DNA sequence variation in bacterial genome evolution and mutagenicity [12, 14–16]. Their mobility in the genome can lead to detrimental, advantageous or neutral effects on the bacteria fitness [17–20]. We previously studied the IS-mediated genome structural variations (SVs) in the selection-free conditions using whole genome re-sequencing data from mutation accumulation (MA) lines of the *Escherichia coli* K12 MG1655 strain [21]. We observed that IS insertions and IS-induced recombinations constitute most of the spontaneous genome SVs events. We reported on average, 3.5×10^−4^ IS insertions and 4.5×10^−5^ IS-mediated recombinations occur spontaneously per genome per generation in the *E. coli* K12 MG1655 genome, and these rates remain constant across the wild-type and 12 DNA repair deficient mutants [21].

An immediate question following up this study is if and how these rates change across different bacterial genomes (different species or different strains of the same species). Shewaramani and colleagues [22] investigated MA lines of *E. coli* REL4536 strain grown aerobically and anaerobically, respectively, and reported that the spontaneous rate of IS insertions is 2.1×10^−4^ per genome per generation when it is grown in an aerobic environment, and is elevated to 6.4×10^−4^ when it is grown in an anaerobic (oxidative stress) environment, both comparable (within two-fold of difference) with the IS insertions rate reported in *E. coli* K12 MG1655 genome [21, 22]. Moreover, our analyses on the IS-mediated structural variations in the MA lines of *Deinococcus radiodurans* BAA-816 wild type strain and *D. radiodurans* R1 (ATCC13949) DNA repair deficient mutant (*mutL* ^−^) revealed much higher rates, 2.5×10^−3^ and 4.8×10^−4^ IS insertions per genome per generation, respectively [23]. Although these observations may suggest that the spontaneous movement of IS elements does not follow a conserved rate in different bacterial genomes, the data are too sparse to be conclusive.

In this study, we conducted a comparative analysis of IS-mediated genome structural variations (SVs) in ten previously published mutation accumulation (MA) experiments [22–27] conducted on eight strains from five bacterial species (Table 1, Additional file 1: Table ST1), which span both gram-negative (i.e., *E. coli*, *Burkholderia cenocepacia* and *Vibrio cholerae*) and gram-positive bacteria (i.e., *Mycobacterium smegmatis* and *D. radiodurans*). Furthermore, the data include four different and divergent strains of *E. coli* (see Additional file 1: Figure SF1): ED1a, IAI1, REL4536 in addition to the *E. coli* K12 MG1655 analyzed previously by us [21]. We used GRASPER [28], a *denovo* structural variation identification algorithm, to identify SVs in each MA experiment, and then identified IS-mediated SVs among them. Note that we conducted the re-analyses of the published datasets using GRASPER so that the results could be directly comparable.

**Table 1:** IS-mediated structural variation rates across bacterial genomes

Bacteria strain	Genome size	Number of	Generations	Insertions		Deletions		Reference
	[Mbps]	MA lines	per MA line	IS-related	Rate [ ×10^−4^]	IS-related	Rate [ ×10^−5^]	
*B. cenocepacia* HI2424	7.70	50	5500	57	2.10			This study
*V. cholerae* 2740-80 MMR mut	4.09	48	1254	1	0.02			This study
*M. smegmatis* MC2 155	6.99	49	4900	106	4.40	202	84.0	This study
*D. radiodurans* BAA-816	3.8	43	5961	640	25.0			[23]
*D. radiodurans* R1 (ATCC13949) mutL-	3.8	19	993	9	4.80			[23]
*E. coli* K12 MG1655	4.64	520	4186	758	3.50	98	4.50	[21]
*E. coli* REL4536 (Aerobic)	4.63	24	4500	58	5.40	78	72.0	This study
*E. coli* REL4536 (Anaerobic)	4.63	24	3456	166	20.0	78	94.0	This study
*E. coli* ED1a	5.21	50	6114	7	0.23	11	3.60	This study
*E. coli* IAI1	4.7	49	6342	-	-	2	0.64	This study

In previous studies, the IS insertion rate was reported to be approximately 2.5×10^−3^ insertions per genome per generation in the wild type of *D. radiodurans* BAA-816, which is much higher than the rate of 4.8×10^−4^ IS insertions per genome per generation observed in *D. radiodurans* R1 (ATCC13949) mismatch repair (MMR)-deficient strain [23], and the rate of 3.5×10^−4^ IS insertions per genome per generation reported in *E. coli* K12 MG1655 [21]. Our results indicate that the insertion and recombination rates of IS elements vary between different bacterial genomes and even among strains of the same species

**Fig. 1:**
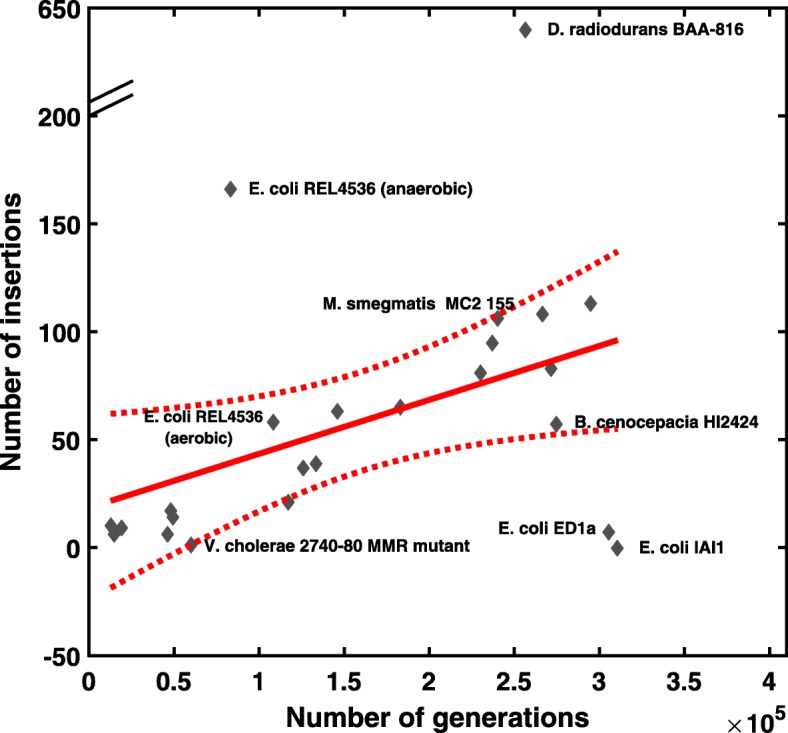
IS insertion rates vary across different bacterial genomes. IS insertion rates of various bacterial genomes are compared to the IS insertion rates in the wild-type and 12 DNA repair deficient mutants of *E. coli* K12 MG1655 reported previously [21]. This figure plotted the number of observed IS insertions (y-axis) versus the total number of generations (x-axis) in a group of MA lines originating from the same founder strain in a single experiment. While all the MA experiments on *E. coli* K12 MG1655 exhibited a linear relationship between the number of insertions and the number of generations, suggesting a constant IS insertion rate per generation across these lines, only in some of the other *E. coli* strains and bacterial species studied here, similar IS insertion rates were observed. In contrast, much higher rates were observed in wild-type *D. radiodurans* BAA-816 and *E. coli* REL4536 grown in the anaerobical condition, whereas much lower rates are observed in *E. coli* ED1a and IAI1 strains.For the linear regression, the dotted line shows the 95% confidence interval boundaries

Our findings indicate that there is a divergence in the rates of IS-mediated insertions and recombinations, both within and among bacterial species (Table 1 and Fig. 1). The insertion rate of IS elements in *M. smegmatis* MC2 155 is approximately 4.4×10^−4^ IS insertions per genome per generation, a rate comparable to the observed rate in *E. coli* K12 MG1655 [21]. However, we observed a lower IS insertion rate of 1.7×10^−5^ IS insertions per genome per generation in *V. cholerae* 2740-80 MMR deficient strain and the rate of 2.1×10^−4^ IS insertions per genome per generation in *B. cenocepacia* HI2424, respectively. We note the observed discrepancy in IS insertion rates is not due to the variation of genome sizes. For example, the *D. radiodurans* BAA-816 genome is slightly shorter than the *E. coli* K12 MG1655 genome (3.8 Mbps vs. 4.6 Mbps), whereas the insertion rate of IS elements in *D. radiodurans* BAA-816 [23] is about 7 times higher than that in *E. coli* K12 MG1655. More interestingly, the insertion rates of IS elements vary significantly among different strains of *E. coli* (Table 1 and Fig. 1). Specifically, we did not observe any IS insertions in *E. coli* IAI1, while only seven were observed in *E. coli* ED1a (yielding an IS insertion rate of 2.3×10^−5^ insertions per genome per generation; see Table 1). Notably, in some cases, we identified more IS-mediated SVs than the original studies, resulting in slightly higher insertion rates of IS elements. For example, we identified 58 and 166 IS insertions in MA lines of *E. coli* REL4536 grown in aerobic and anaerobic conditions, respectively, which are higher than the numbers reported in the original publication (22 and 53 IS insertion events, respectively) [22].

The activities of IS elements in different IS families are not the same in bacterial genomes. The elements in some IS families are active in some bacterial genomes but not in others. IS2, IS3 and IS150, which belong to the IS3 family, along with IS1 and IS5, are the major constant passengers in *E. coli* strains, and they remain active in these genomes. The activity of IS110 elements are only observed in *M. smegmatis* MC2 155 and in *B. cenocepacia* HI2424 genomes. The IS elements in some families were observed to be active only in specific genomes. For example, IS1096, IS6120 and IS1549 elements are involved in genome structural variations in *M. smegmatis* MC2 155, whereas the activities of IS256, IS66 and IS481 elements were observed only in *B. cenocepacia* HI2424 genome. Among the *E. coli* strains, the activities of IS elements are also divergent: the activity of IS1 element was the only observed in *E. coli* ED1a, while the activity of IS2 elements was only observed in *E. coli* K12 MG1655. Although the elements of some common families (e.g., IS3) are detected in all bacterial genomes (see Additional file 1: Table ST2), there is no IS element/family that was found to be active across all these bacterial genomes (see Additional file 1: Table ST3).

We observed all IS-mediated deletions are due to the homologous recombination between two IS elements in bacterial genomes, consistent with our previous study [21]. Similarly, the rate of IS-mediated deletions varies within and across bacterial species as shown in Table 1.

In summary, the results reported here substantiate that IS-mediated SVs vary among different bacterial species and different strains of the same bacterial species voire within IS families. The cause and impact of this divergence in IS activity remains to be explored. Nevertheless, these observations suggest that the activity of IS elements may not be determined by the mere IS composition within host genome, but rather from an evolutionary mechanism orchestrated by both IS elements and their hosts. The distribution and composition of IS elements are quite sparse across bacterial genomes (see Additional file 1: Table ST2). The quest for plausible explanations of this observation has led to several different but not mutually exclusive views of IS maintenance and proliferation within bacterial genomes. Initially, IS elements were considered as DNA parasites, which do not contribute to host fitness, maintained by their ability of self-replication and mainly spread through horizontal gene transfer [29, 30]. However, there are ample evidence that this is not the proper and well-suited view of the state of IS elements in prokaryotes [14]. In fact, some studies argue that IS elements are maintained by neutral selection where both IS elements and their host coexist in a dynamic equilibrium that defines the co-evolution and shapes the architecture of host genomes [3]. Other studies recognize IS elements as sources of genetic diversity and thus contribute to their host fitness by mediating beneficial mutations through natural selection [17–20]. However, a recent study indicated that transposition bursts do not lead to IS persistence in bacterial genomes despite offering occasional beneficial and adaptive mutations to the host genome in the short term [31]. Obviously, more studies are needed to provide evidence supporting the assertions about the state of IS elements in bacterial genomes. Nevertheless, our observations suggest that the forces driving the activity of IS elements are regulated by both IS elements and their hosts, and thus, the mechanism of IS regulation is not only element-specific but also related to the host bacterial species.

## Additional file

Additional file 1 Supplementary materials. (PDF 129 kb)

## Abbreviations

IS: Insertion sequence
MA: Mutation accumulation
MGE: Mobile genetic elements
MMR: Mismatch repair
SV: Structural variation
WGS: Whole genome sequence
