# Supplementary material for: Insertion sequence elements-mediated structural variations in bacterial genomes
Source: Mob DNA. 2018 Aug 29;9:29. doi: 10.1186/s13100-018-0134-3 (PMC6114881; doi:10.1186/s13100-018-0134-3)
Supplement: Supplementary file 1 — Supplementary materials. (PDF 129 kb) [file 13100_2018_134_MOESM1_ESM.pdf]

Supplementary materials

Materials and methods

An MA experiment involves a serial passage of multiple clonal populations (*MA lines*) derived from a single bacterial founder strain, which are subject to consecutive single-individual bottlenecks allowing mutations to accumulate with little to no selective pressure such that the evolution proceeds by close to pure genetic drift [1, 2]. The subsequent whole genome sequencing of the MA lines can reveal the mutations accumulated during the MA process, together with high throughput whole genome sequencing (WGS) of MA lines. MA-WGS strategy enables genome-wide assessment of spontaneous mutations in bacterial genomes [2, 3, 4, 5, 6, 7]. We used the previously published MA data and the specifications of each data set analyzed in this study are provided in Table ST1 or referred to the original study for more details.

Bacteria strain	Bioproject	SRA study	MA lines	Generations per MA line	Reference genome
B. cenocepacia HI2424	PRJNA13918	SRP003516	50	5500	GCA_000203955.1:<br>NC_008542.1<br>NC_008543.1<br>NC_008544.1<br>NC_008545.1
V. cholerae 2740-80 MMR mut	PRJNA256339	SRP077572	48	1254	GCF_001683415.1:<br>CP016324.1<br>CP016325.1
M. smegmatis MC2 155	PRJNA320082	SRP074205	50	4900	NC_008596.1
D. radiodurans BAA-816<br>*D. radiodurans* R1 (ATCC13949)	PRJNA397203	SRP114859			see [3]
E. coli K12 MG1655	PRJNA168337	SRP013707	520	4186	see [4]
E. coli REL4536 (AE)<br>*E. coli* REL4536 (ANAE)	PRJNA315479	SRP073250	24<br>24	4500<br>3456	*E. coli* REL606, NC_012967.1
E. coli ED1a<br>*E. coli* IAI	PRJNA168337	SRP013707	50<br>49	6114<br>6342	NC_011745.1<br>NC_011741.1

Table ST1: Details about accession numbers of the previously published MA data

The bacterial species in these MA studies span both gram-negative (i.e., *E. coli*, *B. cenocepacia* and *V. cholerae*) and gram-positive bacteria (i.e.,*M. smegmatis* and *D. radiodurans*). The data include four different and divergent strains of *E. coli* namely ED1a, IAI1, REL4536 in addition to the *E. coli* K12 MG1655 MG1655. *E. coli* ED1a and IAI1 are human commensal and recently isolated [8], belonging to the phylogenetic group B2 (ED1a) [9] and B1 (IAI1) [8, 10, 11], respectively. *E. coli* REL4536 is a descendent (10^4 generations) of *E. coli* REL606 [12, 13]. These four *E. coli* strains are divergent, and only share nearly 3000 genes (see Figure SF1).

M. smegmatis MC2 155 is a non-pathogenic and a saprophytic Gram-positive bacteria [14], which often used to study the nefarious and pathogenic *Mycobacterial* species such as *Mycobacterium tuberculosis* [15]. The most characteristic feature of *M. smegmatis* MC2 155 is that it is naturally devoid of post-replicative mismatch repair (MMR) pathway [16].

Many bacteria like *E. coli* carry a single chromosome. Some bacteria contains one or more plasmids inside their cells, whose genomes are also sequenced when the whole genome sequences are characterized. Among the bacterial genomes we analyzed, four carry plasmids: *D. radiodurans* known for its resistance to radiation, oxidation and desiccation [3, 17], *B. cenocepacia* HI2424 known as a clinically relevant pathogen with high GC content [18], and *V. cholerae* 2740-80 which is a notorious pathogen of cholera disease. The two strains of *D. radiodurans* namely R1 and BAA-816

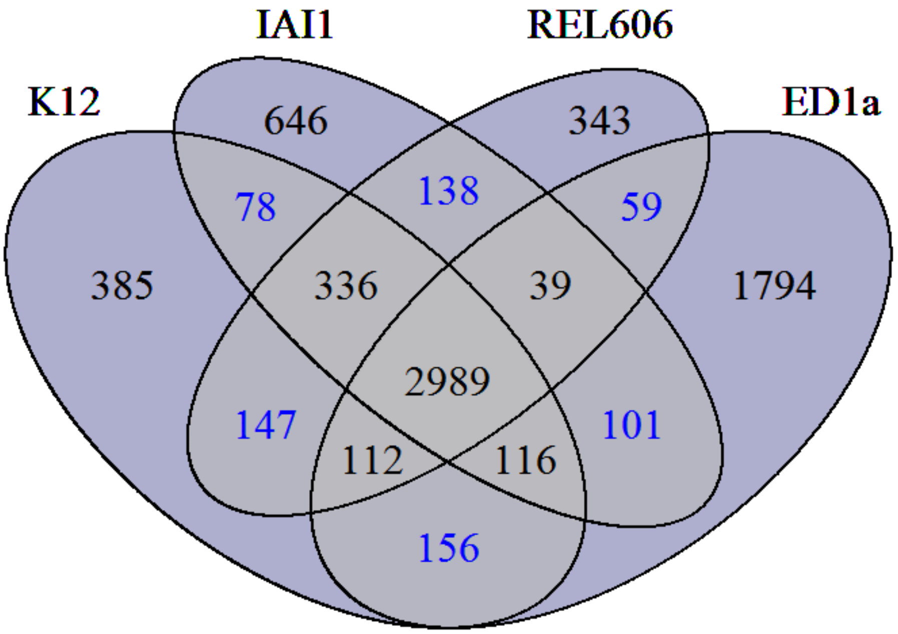

Figure SF1: The count of genes shared between the four strains of *E. coli*: K12 MG1655, ED1a , IAI1 and REL606 indicate that the four *E. coli* strains are different and have diverged significantly.

strains originate from a common culture and remain genetically very similar as there are very few genetic differences between the two strains [3, 19].

Detection of structural variation events

We used the GRASPER algorithm which detects SVs by using an $A_l - Bruijn$ graph representation of the reference genome and paired-end whole-genome sequencing data [4, 5]. GRASPER obtains clusters of discordant read pairs and annotates the SV events as inversions, deletions, transpositions and tandem duplications. GRASPER has been shown to be robust in detecting genomic rearrangements in bacterial MA lines [4]. Our analyses focused mainly on the IS transposition events and other IS-mediated SVs.

Identification of IS-mediated structural variation events

To detect IS-mediated SVs, for every bacterial genome, the respective reference genome annotation file was used to identify IS elements in the genome. In some cases, we used ISfinder database [20] to define IS families for those annotated IS elements without specific assignment of IS families.

The complete list of IS elements in the reference genome of the bacteria strains is provided in the following Table ST2.

GRASPER detects both IS-mediated and non-IS related SV events, from which we identified the IS-mediated SVs based on the genome annotations of IS elements. A transposition event is IS-mediated if the clusters defining the donor span an annotated IS element, while a deletion (or a recombination event) is defined to be IS-mediated if its breakpoint is within a certain distance (default 1000bps) from an annotated IS element. Next, the detected SV events were manually curated to filter out those less likely to be IS-mediated. Further analysis was done to determine the overall rate μ of SVs (transpositions or deletions/recombinations) based on the identified SVs over all the MA lines using the formula $\mu = \frac{n}{m \times g}$, where n is the number of identified IS-mediated SVs, m is the total number of MA lines and g is the average number of generations per MA line. Finally, we combined with the findings from [3, 4, 12] to compare the activity of IS elements across different bacterial genomes.

Variations of insertion rates in different IS families

In our comparative study, we observed that the activities of the IS elements in different IS families are not the same in bacterial genomes; the elements in some IS families are active in some bacterial genomes but not in others. As shown in Table ST3, IS1,IS2, IS3, IS150 and IS5 are the major active IS elements in *E. coli* strains. However, among the *E. coli* strains, the activities of IS elements are divergent: the activity of IS1 elements is only observed in ED1a, while the activity of IS2 elements was only observed in *E. coli* K12 MG1655. The activity of IS110 elements are only observed in the *M. smegmatis* MC2 155 and *B. cenocepacia* HI2424 genomes. The IS elements in some families were observed to be active only in specific genomes. For examples, IS1096, IS6120 and IS1549 elements are involved in genome rearrangements in the *M. smegmatis* MC2 155, whereas the activities of IS256, IS66 and IS481 elements were observed only in the *B. cenocepacia* HI2424 genome. There is no IS element/family that was found to be active across all these bacterial genomes (Table ST3), although the elements of some common families (e.g., IS3) are detected in all bacterial genomes.

IS element	*B. cenocepacia* HI2424	*V.cholerae* 2740-80	*M. smegmatis* MC2 155	*D. radiodurans* BAA-816	*E. coli* K12 MG1655	*E. coli* REL606	*E. coli* ED1a	*E. coli* IAI1
IS009						2		
IS061						2		
IS1					7	55	25	2
IS102						2		
IS1096			48					
IS110	6		4				5	7
IS1137			5					
IS116/IS110/IS902			3					
IS128						2		
IS150					3	9		
IS1549			5					
IS186					3	5		
IS186A					1			
IS186B					1			
IS186C					1			
IS1A					1			
IS1B					1			
IS1C					1			
IS1D					1			
IS1E					1			
IS1F					1			
IS1H					1			
IS1I					1			
IS2					6	1		
IS200/IS605	7	6					3	5
IS21	3		2				2	
IS256	2		2					
IS2621				10				
IS2A					1			
IS2D					1			
IS2E					1			
IS2F					1			
IS2H					1			
IS2I					1			
IS2K					1			
IS3	13	3	16		11	5	19	10
IS30						1		
IS30	1				4			
IS30A					1			
IS30B					1			
IS30C					1			
IS30D					1			
IS3A					1			
IS3B					1			
IS3C					1			
IS3D					1			
IS3E					1			
IS4					2	1		1
IS481	3	2	1					1
IS481	3							
IS5	6	1			11		1	
IS5/IS1182	2							
IS5A					1			
IS5B					1			
IS5D					1			
IS5F					1			
IS5H					1			
IS5I					1			
IS5LO					1			
IS5R					1			
IS5T					1			
IS5U					1			
IS5Y					1			
IS600					1	3		
IS609					3			
IS6120			2					
IS66	5						9	1
IS91			2				1	
IS911					5	2		
IS911A					2			
IS911B					2			
ISAs1		1					1	1
ISDra				1				
ISDra1				9				
ISDra2				8				
ISDra3				2				
ISDra4				4				
ISDra5				2				
ISDra6				3				
ISL3	1							
ISL3							1	
ISMsm1			5					
ISMsm2			4					
ISMsm3			2					
ISMsm4			4					
ISMsm5			5					
ISMsm6			1					
ISMsm7			2					
ISMsm8			2					
ISNCY	2						1	2
ISRSO11						1		
ISX					1			
ISZ					1			
Tn3	2							

Table ST2: IS element copies found in the reference genomes of the bacterial species analyzed in this study. The IS copy number was derived from the annotation file and ISFinder [20] database when the IS element was annotated without specific name. The distribution of IS elements is very sparse and different across bacterial species.

IS element	*B. cenocepacia*	*V. cholerae*	*M. smegmatis*	*D. radiodurans*		*E. coli*				
	HI2424	2740-80 MMR mut	MC2 155	BAA-816	R1 (ATCC13949) mutL$^-$	K12 MG1655	REL4536 (AE)	REL4536 (ANAE)	ED1a	IAI
IS1				2.93×10^{-4}		1.08×10^{-4}	4.63×10^{-5}	4.58×10^{-4}	1.96×10^{-5}	
IS1096	8.75×10^{-5}									
IS110			7.27×10^{-6}							
IS116/IS110/IS902			2.79×10^{-4}							
IS150						9.19×10^{-7}	5.56×10^{-5}	4.82×10^{-4}		
IS1549			6.25×10^{-5}							
IS186						3.12×10^{-5}	8.33×10^{-5}	4.70×10^{-4}		
IS2						1.38×10^{-6}				
IS200/IS605				1.25×10^{-4}						
IS256	7.27×10^{-6}									
IS3	1.71×10^{-4}		4.16×10^{-6}			3.26×10^{-5}	1.76×10^{-4}	4.58×10^{-4}		
IS4				2.03×10^{-4}	1.59×10^{-4}	3.68×10^{-6}		1.21×10^{-5}		
IS481	3.64×10^{-6}									
IS5						1.67×10^{-4}		3.62×10^{-5}		
IS5/IS1182	1.09×10^{-5}									
IS600							2.78×10^{-5}	6.03×10^{-5}		
IS609							1.20×10^{-4}	2.65×10^{-4}		
IS6120			2.79×10^{-4}							
IS630				1.88×10^{-3}	3.18×10^{-4}					
IS66	7.27×10^{-6}									
IS911							6.48×10^{-5}	6.03×10^{-5}		
ISMsm4			4.16×10^{-6}							

Table ST3: IS elements transposition rates across bacterial genomes. The activities of IS families are divergent across bacterial species as well as across the strains of the same species.

References

[1] Daniel L. Halligan and Peter D. Keightley. Spontaneous mutation accumulation studies in evolutionary genetics. *Annual Review of Ecology, Evolution, and Systematics*, 40(1):151–172, 2009.

[2] F. A. Kondrashov and A. S. Kondrashov. Measurements of spontaneous rates of mutations in the recent past and the near future. *Philos. Trans. R. Soc. Lond., B, Biol. Sci.*, 365(1544):1169–1176, Apr 2010.

[3] Hongan Long, Sibel Kucukyildirim, Way Sung, Emily Williams, Heewook Lee, Matthew Ackerman, Thomas G. Doak, Haixu Tang, and Michael Lynch. Background mutational features of the radiation-resistant bacterium deinococcus radiodurans. *Molecular Biology and Evolution*, 32(9):2383, 2015.

[4] Heewook Lee, Thomas G Doak, Ellen Popodi, Patricia L Foster, and Haixu Tang. Insertion sequence-caused large-scale rearrangements in the genome of escherichia coli. *Nucleic acids research*, 44(15):7109–7119, 2016.

[5] Heewook Lee, Ellen Popodi, Patricia L Foster, and Haixu Tang. Detection of structural variants involving repetitive regions in the reference genome. *Journal of Computational Biology*, 21(3):219–233, 2014.

[6] Patricia L. Foster, Heewook Lee, Ellen Popodi, Jesse P. Townes, and Haixu Tang. Determinants of spontaneous mutation in the bacterium escherichia coli as revealed by whole-genome sequencing. *Proceedings of the National Academy of Sciences*, 112(44):E5990–E5999, 2015.

[7] Patricia L Foster, Heewook Lee, Ellen Popodi, Jesse P Townes, and Haixu Tang. Determinants of spontaneous mutation in the bacterium escherichia coli as revealed by whole-genome sequencing. *Proceedings of the National Academy of Sciences*, 112(44):E5990–E5999, 2015.

[8] M. Touchon, C. Hoede, O. Tenaillon, V. Barbe, S. Baeriswyl, P. Bidet, E. Bingen, S. Bonacorsi, C. Bouchier, O. Bouvet, A. Calteau, H. Chiapello, O. Clermont, S. Cruveiller, A. Danchin, M. Diard, C. Dossat, M. E. Karoui, E. Frapy, L. Garry, J. M. Ghigo, A. M. Gilles, J. Johnson, C. Le Bouguenec, M. Lescat, S. Mangenot, V. Martinez-Jehanne, I. Matic, X. Nassif, S. Oztas, M. A. Petit, C. Pichon, Z. Rouy, C. S. Ruf, D. Schneider, J. Tourret, B. Vacherie, D. Vallenet, C. Medigue, E. P. Rocha, and E. Denamur. Organised genome dynamics in the Escherichia coli species results in highly diverse adaptive paths. *PLoS Genet.*, 5(1):e1000344, Jan 2009.

[9] H. Toh, K. Oshima, A. Toyoda, Y. Ogura, T. Ooka, H. Sasamoto, S. H. Park, S. Iyoda, K. Kurokawa, H. Morita, K. Itoh, T. D. Taylor, T. Hayashi, and M. Hattori. Complete genome sequence of the wild-type commensal Escherichia coli strain SE15, belonging to phylogenetic group B2. *J. Bacteriol.*, 192(4):1165–1166, Feb 2010.

[10] M. Touchon, C. Hoede, O. Tenaillon, V. Barbe, S. Baeriswyl, P. Bidet, E. Bingen, S. Bonacorsi, C. Bouchier, O. Bouvet, A. Calteau, H. Chiapello, O. Clermont, S. Cruveiller, A. Danchin, M. Diard, C. Dossat, M. E. Karoui, E. Frapy, L. Garry, J. M. Ghigo, A. M. Gilles, J. Johnson, C. Le Bouguenec, M. Lescat, S. Mangenot, V. Martinez-Jehanne, I. Matic, X. Nassif, S. Oztas, M. A. Petit, C. Pichon, Z. Rouy, C. S. Ruf, D. Schneider, J. Tourret, B. Vacherie, D. Vallenet, C. Medigue, E. P. Rocha, and E. Denamur. Organised genome dynamics in the Escherichia coli species results in highly diverse adaptive paths. *PLoS Genet.*, 5(1):e1000344, Jan 2009.

[11] O. Lukjancenko, T. M. Wassenaar, and D. W. Ussery. Comparison of 61 sequenced Escherichia coli genomes. *Microb. Ecol.*, 60(4):708–720, Nov 2010.

[12] Sonal Shewaramani, Thomas J. Finn, Sinead C. Leahy, Rees Kassen, Paul B. Rainey, and Christina D. Moon. Anaerobically grown escherichia coli has an enhanced mutation rate and distinct mutational spectra. *PLOS Genetics*, 13(1):1–22, 01 2017.

[13] H. Jeong, V. Barbe, C. H. Lee, D. Vallenet, D. S. Yu, S. H. Choi, A. Couloux, S. W. Lee, S. H. Yoon, L. Cattolico, C. G. Hur, H. S. Park, B. Segurens, S. C. Kim, T. K. Oh, R. E. Lenski, F. W. Studier, P. Daegelen, and J. F. Kim. Genome sequences of Escherichia coli B strains REL606 and BL21(DE3). *J. Mol. Biol.*, 394(4):644–652, Dec 2009.

[14] R. E. GORDON and M. M. SMITH. Rapidly growing, acid fast bacteria. I. Species' descriptions of Mycobacterium phlei Lehmann and Neumann and Mycobacterium smegmatis (Trevisan) Lehmann and Neumann. *J. Bacteriol.*, 66(1):41–48, Jul 1953.

[15] M. Altaf, C. H. Miller, D. S. Bellows, and R. O'Toole. Evaluation of the Mycobacterium smegmatis and BCG models for the discovery of Mycobacterium tuberculosis inhibitors. *Tuberculosis (Edinb)*, 90(6):333–337, Nov 2010.

[16] Sibel Kucukyildirim, Hongan Long, Way Sung, Samuel F. Miller, Thomas G. Doak, and Michael Lynch. The rate and spectrum of spontaneous mutations in mycobacterium smegmatis, a bacterium naturally devoid of the postreplicative mismatch repair pathway. *G3: Genes, Genomes, Genetics*, 6(7):2157–2163, 2016.

[17] V. K. Charaka and H. S. Misra. Functional characterization of the role of the chromosome I partitioning system in genome segregation in Deinococcus radiodurans. *J. Bacteriol.*, 194(21):5739–5748, Nov 2012.

[18] Marcus M. Dillon, Way Sung, Michael Lynch, and Vaughn S. Cooper. The rate and molecular spectrum of spontaneous mutations in the gc-rich multichromosome genome of burkholderia cenocepacia. *Genetics*, 200(3):935–946, 2015.

[19] O. White, J. A. Eisen, J. F. Heidelberg, E. K. Hickey, J. D. Peterson, R. J. Dodson, D. H. Haft, M. L. Gwinn, W. C. Nelson, D. L. Richardson, K. S. Moffat, H. Qin, L. Jiang, W. Pamphile, M. Crosby, M. Shen, J. J. Vamathevan, P. Lam, L. McDonald, T. Utterback, C. Zalewski, K. S. Makarova, L. Aravind, M. J. Daly, K. W. Minton, R. D. Fleischmann, K. A. Ketchum, K. E. Nelson, S. Salzberg, H. O. Smith, J. C. Venter, and C. M. Fraser. Genome sequence of the radioresistant bacterium Deinococcus radiodurans R1. *Science*, 286(5444):1571–1577, Nov 1999.

[20] Lestrade L Mahillon J Chandler M. Siguier P, Perochon J. Isfinder: the reference centre for bacterial insertion sequences. *Nucleic acids research*, 34.
